# QTL Analysis of Z414, a Chromosome Segment Substitution Line with Short, Wide Grains, and Substitution Mapping of *qGL11* in Rice

**DOI:** 10.1186/s12284-022-00571-7

**Published:** 2022-05-09

**Authors:** Juan Li, Hongxia Yang, Guangyi Xu, Keli Deng, Jinjin Yu, Siqian Xiang, Kai Zhou, Qiuli Zhang, Ruxiang Li, Miaomiao Li, Yinghua Ling, Zhenglin Yang, Guanghua He, Fangming Zhao

**Affiliations:** Rice Research Institute, Academy of Agricultural Sciences, Southwest University, Chongqing, 400715 China

**Keywords:** Rice, Chromosome segment substitution line, Grain size, Quantitative trait locus, Substitution mapping, *qGL11*

## Abstract

Most agronomic traits of rice (*Oryza sativa*), such as grain length, are complex traits controlled by multiple genes. Chromosome segment substitution lines (CSSLs) are ideal materials for dissecting these complex traits. We developed the novel rice CSSL ‘Z414’, which has short, wide grains, from progeny of the recipient parent ‘Xihui 18’ (an *indica* restorer line) and the donor parent ‘Huhan 3’ (a *japonica* cultivar). Z414 contains four substitution segments with an average length of 3.04 Mb. Z414 displays seven traits that significantly differ from those of Xihui 18, including differences in grain length, width, and weight; degree of chalkiness; and brown rice rate. We identified seven quantitative trait loci (QTL) that are responsible for these differences in an F_2_ population from a cross between Xihui 18 and Z414. Among these, six QTL (*qPL3*, *qGW5*, *qGL11*, *qRLW5*, *qRLW11*, and *qGWT5*) were detected in newly developed single-segment substitution lines (SSSLs) S1–S6. In addition, four QTL (*qGL3*, *qGL5*, *qCD3*, and *qCD5*) were detected in S1 and S5. Analysis of these SSSLs attributed the short, wide grain trait of Z414 to *qGL11*, *qGL3*, *qGL5*, and *qGW5.* Substitution mapping delimited *qGL11* within an 810-kb interval on chromosome 11. Sequencing, real time quantitative PCR, and cell morphology analysis revealed that *qGL11* might be a novel QTL encoding the cyclin CycT1;3. Finally, pyramiding *qGL3* (a = 0.43) and *qGL11* (a =  − 0.37) led to shorter grains in the dual-segment substitution line D2 and revealed that *qGL11* is epistatic to *qGL3*. In addition, S1 and D2 exhibited different grain sizes and less chalkiness than Z414. In conclusion, the short grain phenotype of the CSSL Z414 is controlled by *qGL11*, *qGL3*, and *qGL5*. *qGL11* might be a novel QTL encoding CycT1;3, whose specific role in regulating grain length was previously unknown, and *qGL11* is epistatic to *qGL3*. S1 and D2 could potentially be used in hybrid rice breeding.

**Supplementary Information:**

The online version contains supplementary material available at 10.1186/s12284-022-00571-7.

## Background

Grain size and weight determine rice yield and quality (Feng et al. 2021). To date, more than 400 quantitative trait loci (QTL) related to grain size have been mapped (Huang et al. 2013) and some of these have been cloned. Several signaling pathways determining grain size have been identified, including phytohormone signaling pathways, G protein signaling pathways, the ubiquitin–proteasome pathway, mitogen-activated protein kinase (MAPK) signal transduction, and pathways regulated by transcription factors (Li and Li 2016).

Several phytohormones are important in determining rice grain size, including brassinosteroids (BRs) and auxins (indole-3-acetic acid; IAA). Three QTL for grain size might be involved in BR signaling: *Grain size 5* (*GS5*) encodes a putative serine carboxypeptidase that competitively inhibits the interaction between BRI 1-ASSOCIATED RECEPTOR KINASE 1 (OsBAK1-7) and MEMBRANE STEROID-BINDING PROTEIN 1 (OsMSBP1) thus influencing BR signaling. Higher *GS5* expression levels result in wide, heavy grains caused by increased cell proliferation and expansion in spikelet hulls (Li et al. 2011; Xu et al. 2015). *QTL for grain length 3.1* (*GL3.1*) suppresses BR signaling by regulating the phosphorylation and stability of *GLYCOGEN SYNTHASE KINASE 3 (GSK3)/SHAGGY-LIKE KINASE 3* (*OsGSK3*) (Gao et al. 2019). *QTL for grain width and weight on chromosome 5* (*GW5*) encodes a positive regulator of BR signaling that represses GSK2 kinase activity, resulting in the accumulation of unphosphorylated *Oryza sativa* BRASSINAZOLE RESISTANT 1 (*Os*BZR1) and DWARF AND LOW-TILLERING (DLT) proteins in the nucleus to mediate BR-responsive gene expression and growth responses, thus affecting grain width (Liu et al. 2017).

Two QTL for grain size might participate in IAA signaling. *Thousand-grain weight 6* (*TGW6*) encodes IAA-glucose hydrolase. In sink organs, the Nipponbare *tgw6* allele limits cell number and grain length by controlling the IAA supply. Loss of function of the Kasalath allele significantly enhances grain weight and yield (Ishimaru et al. 2013). *Big grain 1* (*BG1*) controls grain size by positively regulating IAA responses and transport (Liu et al. 2015a).

G protein signaling is also involved in regulating grain size. In G protein complexes, the Gα subunit RICE G PROTEIN Α SUBUNIT 1 (RGA1) is important for grain expansion, and the Gβ subunit RICE G PROTEIN Β SUBUNIT 1 (RGB1) is essential for plant survival and growth. The Gγ subunit GS3 also acts as a brake in this pathway, reducing grain length by competing for binding to RGB1 to inhibit the downstream signaling of the Gγ subunits Dense and Erect Panicle 1 (DEP1) and ATYPICAL Gγ PROTEIN (GGC2) (Sun et al. 2018).

Ubiquitin can directly or indirectly regulate grain size by affecting protein transport, signal transduction, and protein degradation. *QTL for grain width and weight 2* (*GW2*) encodes a RING-type E3 ubiquitin ligase that negatively regulates cell division by targeting its substrate to the proteasome for proteolysis. A deletion of 1 bp in the *GW2* allele from rice cultivar WY3 causes premature termination of translation. This prevents substrates that should be degraded from being recognized, leading to increased grain length (Song et al. 2007). *Large grain 1* (*LG1*) encodes UBIQUITIN-SPECIFIC PROTEASE 15 (OsUBP15), which possesses de-ubiquitination activity in vitro. The loss of function or downregulation of *OsUBP15* leads to smaller grains (Shi et al. 2019).

MAPK cascades transmit developmental signals to their target molecules via sequential phosphorylation (Xu and Zhang 2015). *OsMKKK10*, *OsMKK4*, and *OsMAPK6* act in the same MAPK pathway to positively regulate grain size (Xu et al. 2018). *Grain Size and Number 1* (*GSN1*) is a negative regulator of the *OsMKKK10–OsMKK4–OsMPK6* cascade; the *gsn1* mutant has a larger grain size than the wild type (Guo et al. 2018).

Transcription factors are also important in controlling seed size. *Grain size 2* (*GS2*) affects grain size by encoding the transcription factor OsGRF4 (Duan et al. 2015). *Grain weight 8* (*GW8*) encodes an SBP domain-containing transcription factor that regulates grain width by binding to the *Grain weight 7* (*GW7*) promoter and repressing its expression (Wang et al. 2015).

Although several genes for grain size in rice have been cloned, our understanding of the mechanisms regulating rice grain size is incomplete. Thus, it is important to identify additional QTL for grain size. Here, we identified the chromosome segment substitution line (CSSL) ‘Z414’, with short, wide grains. Z414 contains four substitution segments from the *indica* restorer line ‘Xihui 18’ as the recipient parent and ‘Huhan 3’ as the donor parent and addressed the following three questions: (1) how many QTL affect the traits in this line and how are they distributed in the four substitution segments? (2) If more than one QTL controls the same trait in Z414, do they exhibit independent inheritance or epistatic interactions? (3) Which of these QTL are novel?

To answer these questions, we systematically characterized Z414 and mapped QTL for associated traits in a secondary F_2_ population derived from a cross between Xihui 18 and Z414. We validated these QTL and analyzed the inheritance model and pyramid effects of target QTL using single-segment substitution lines (SSSLs) and dual-segment substitution lines (DSSLs) developed in the F_3_ generation. Finally, we identified candidate genes of the major QTL *qGL11*. Our findings will be important for breeding by design in rice.

## Results

### Identification of Substitution Segments and Phenotypic Analysis of Z414

**Fig. 1:**
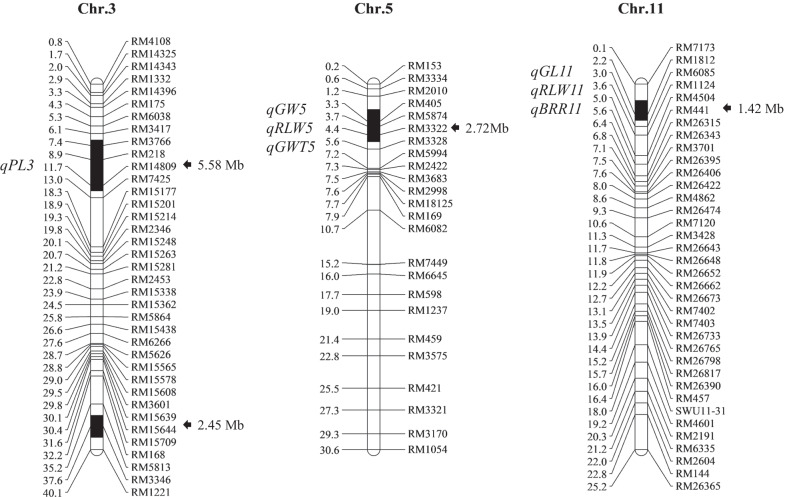
Chromosome substitution segments of Z414. Physical distances (Mb) and mapped QTL are indicated on the left; markers and substitution segment lengths are shown on the right. The black sections on each chromosome are substitution segments. PL, panicle length; GW, grain width; GL, grain length; RLW, ratio of length to width; GWT, 1000-grain weight; BRR, brown rice rate

In this study, we used eight polymorphic simple sequence repeat (SSR) markers in the substitution segments of Z414, and 233 polymorphic SSR markers outside the substitution segments of this line to examine the molecular background of Z414. The substitution segments of 10 Z414 plants were consistent, and no other residual segments from Huhan 3 were detected. Z414 contains four substitution segments from Huhan 3, which are on chromosomes 3, 5, and 11. The total estimated length of the substitution segments was 12.17 Mb, and the average length was 3.04 Mb (Fig. 1).

**Fig. 2:**
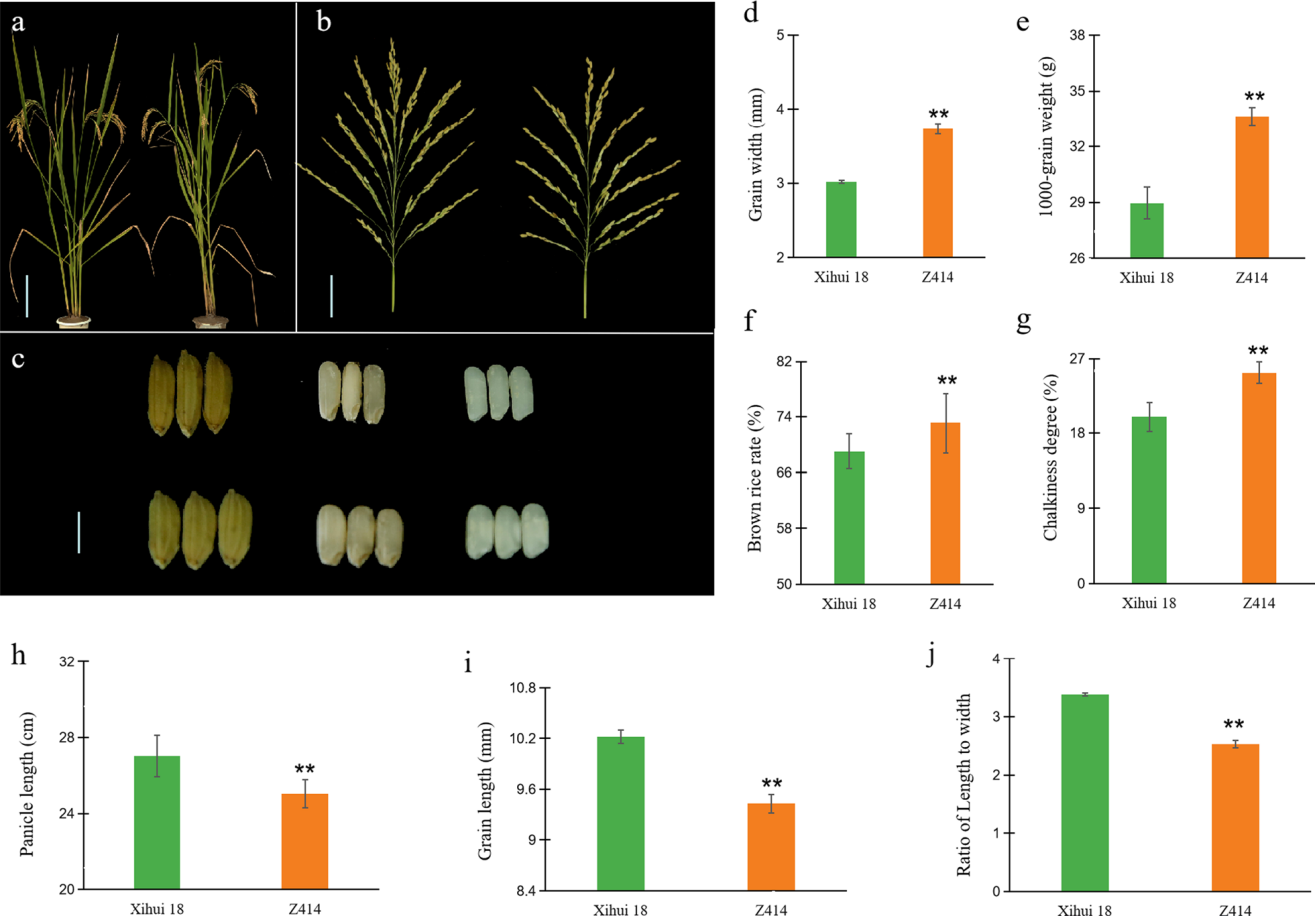
Phenotypes of Xihui 18 and Z414. **a** Plant types of Xihui 18 and Z414. **b** Panicles of Xihui 18 and Z414. **c** Grains, brown rice, and polished rice of Xihui 18 and Z414. Bars represent 20 cm in **a**, 5 cm in **b**, and 5 mm in **c**. **d**–**j** Statistical analysis of the differences in seven traits between Z414 and Xihui 18, in order as grain width (**d**), 1000-grain weight (**e**), brown rice rate (**f**), chalkiness degree (**g**), panicle length (**h**), grain length (**i)**, ratio of grain length to width (**j**). * and ** indicate significant differences at the 0.05 and 0.01 level, respectively

Z414 displayed a similar plant type and different grain size to Xihui 18 (Fig. 2a–c). There were significant increases in grain width (+ 23.5%; Fig. 2d), 1000-grain weight (+ 16.4%; Fig. 2e), brown rice rate (+ 8.8%; Fig. 2f), and degree of chalkiness (+ 26.1%; Fig. 2g). By contrast, Z414 showed significant decreases in panicle length (− 17.5%; Fig. 2h), grain length (− 7.7%; Fig. 2i), and the ratio of length to width (− 25.1%; Fig. 2j). There were no significant differences in other traits, such as plant height, panicle number per plant, spikelet number per panicle, grain number per panicle, seed-setting rate, yield per plant, head rice rate, chalky rice rate, and gel consistency.

### Cytological Analysis of Z414 and Xihui 18 Glumes

**Fig. 3:**
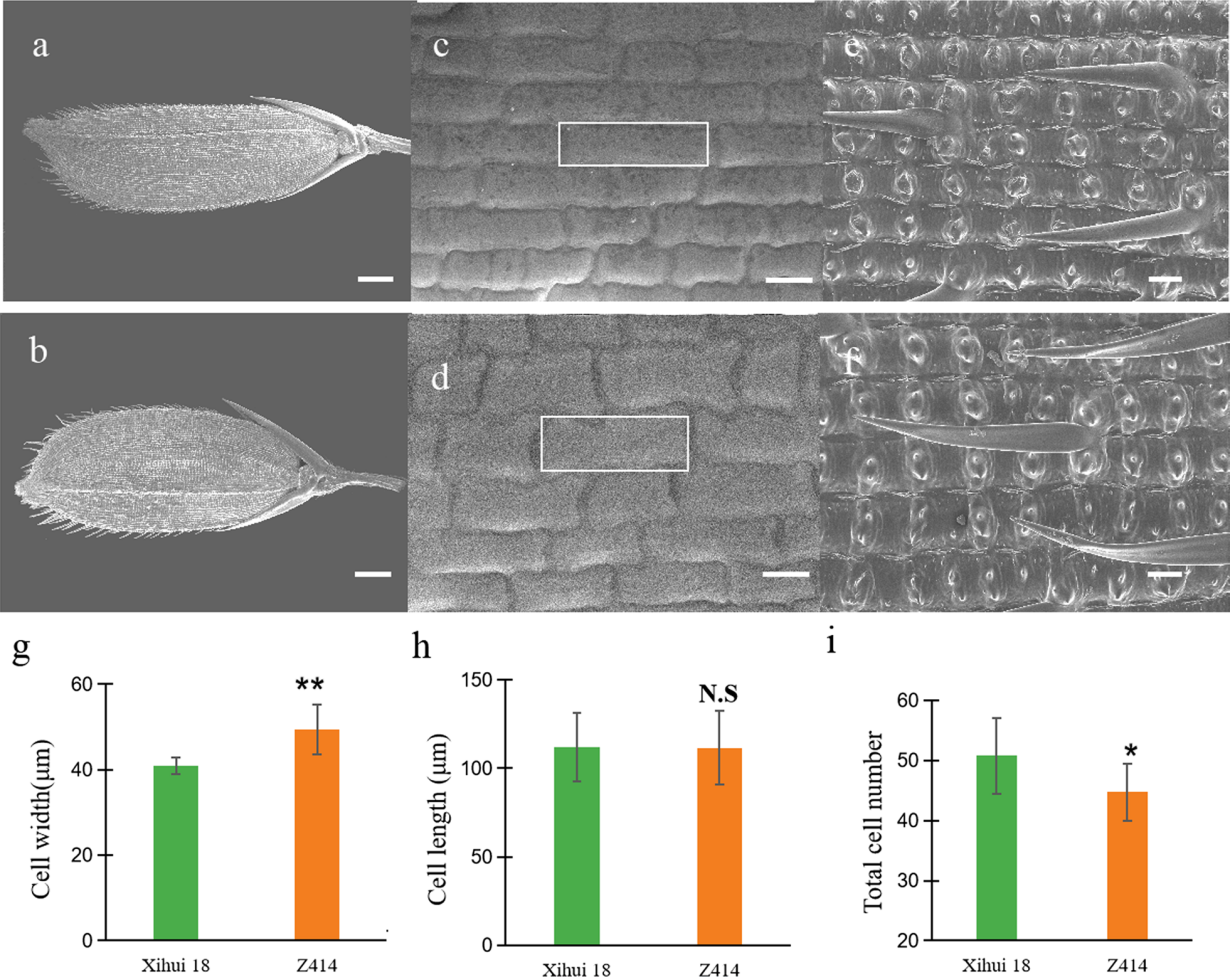
Scanning electron microscopy of Xihui 18 and Z414 glumes. **a**–**f** Scanning electron micrographs of the lemma (**a**, **b**), inner epidermis (**c**, **d**) and outer epidermis (**e**, **f**) of Xihui 18 (**a**, **c**, **e**) and Z414 (**b**, **d**, **f**) glumes. **g**, **h**, **i** show cell width, cell length, and total number of cells in the outer epidermis of the lemma at 200× magnification, respectively. ** and * indicate significant differences at the 0.01 and 0.05 level between Xihui 18 and Z414, respectively. Bars represent 10 mm in **a** and **b**, 500 µm in **c** and **d**, and 500 µm in **e** and **f**

Since the grain lengths and widths differed between Xihui 18 and Z414 (Fig. 3a, b), we used scanning electron microscopy (SEM) to analyze the cell morphology of Xihui 18 and Z414 glumes at the heading stage (Fig. 3c–f). The cell width in the inner epidermis of the glumes was significantly higher in Z414 than in Xihui 18 (+ 22.23%; Fig. 3g). There was no significant difference between Z414 and Xihui 18 in cell length of the inner glume epidermis (Fig. 3h). The total cell number in the outer epidermis of the glume along the longitudinal axis was significantly lower in Z414 than in Xihui 18 (− 13.52%; Fig. 3i). These results indicate that the short, wide grains of Z414 are mainly attributed to a decrease in glume cell number and an increase in glume cell width.

### Identification of QTL Using a Secondary F_2_ Population from Xihui 18/Z414

Seven QTL were identified for six traits that differed between Z414 and Xihui 18, which explained 7.00–42.50% of the phenotypic variation (Table 1). According to the setting of mapQTL, the positive genetic effect of each QTL indicated that the allele of Huhan 3 substitution segment of Z414 increased the phenotypic value, whereas the negative effect indicated that the allele of Xihui18 in substitution interval increased the phenotypic value. Thus, the additive effect of the allele *qGW5* from a Huhan 3 substitution segment of Z414 increased grain width by 0.16 mm, and the dominant effect of *qGW5*/*qgw5* was 0.0004, explaining 42.50% of the phenotypic variance. The additive effect of the *qGWT5* allele from the Huhan 3 substitution segment of Z414 increased the 1000-grain weight by 1.25 g, and the dominant effect of *qGWT5*/*qgwt5* was 0.15. Furthermore, *qGW5*, *qRLW5*, and *qGWT5* were all linked to the same marker, RM5874. The additive of the allele *qGL11* from Xihui 18 in the substitution interval increased grain length by 0.09 mm, and the dominant effect of *qGL11/qgl11* was 0.012, explaining 9.61% of the phenotypic variance in grain length. Similarly, *qGL11, qRLW11* and *qBRR11* were all linked to the same marker, RM1812. However, the additive effects of alleles *qGL11* and *qRLW11* from Xihui 18 in the substitution interval increased the values of the corresponding traits, whereas the *qBRR11* allele from the Huhan 3 substitution segment of Z414 increased the phenotypic value (Fig. 1, Table 1). In addition, the additive effect of the *qPL3* allele from Xihui18 in the substitution interval increased panicle length of Z414 by 1.17 cm per panicle, and the dominant effect of *qPL3*/*qpl3* was 0.59 (Table 1).

**Table 1:** QTL for agronomic and quality traits identified in substitution segments of Z414

Trait	QTL	Chr.	Near marker	A	D	PVE (%)	LOD
Panicle length (cm)	*qPL3*	3	RM3766	− 1.17	+ 0.59	20.30	6.41
Grain width (mm)	*qGW5*	5	RM5874	+ 0.16	+ 0.004	42.50	15.62
Grain length (mm)	*qGL11*	11	RM1812	− 0.09	+ 0.012	9.61	3.07
Ratio of length to width	*qRLW5*	5	RM5874	− 0.15	+ 0.008	40.90	14.84
Ratio of length to width	*qRLW11*	11	RM1812	− 0.06	+ 0.06	10.70	3.21
1000-grain weight (g)	*qGWT5*	5	RM5874	+ 1.25	+ 0.15	20.20	6.38
Brown rice rate (%)	*qBRR11*	11	RM1812	+ 1.44	+ 0.84	7.00	2.76

+ indicates that allele from Huhan3 substitution segment of Z414 increases the trait value, and − indicates that Xihui18 allele in substitution interval increases the trait value. PVE indicates phenotypic variance explained

### Verification and Pyramiding of QTL Using the Newly Developed SSSLs and DSSLs

**Fig. 4:**
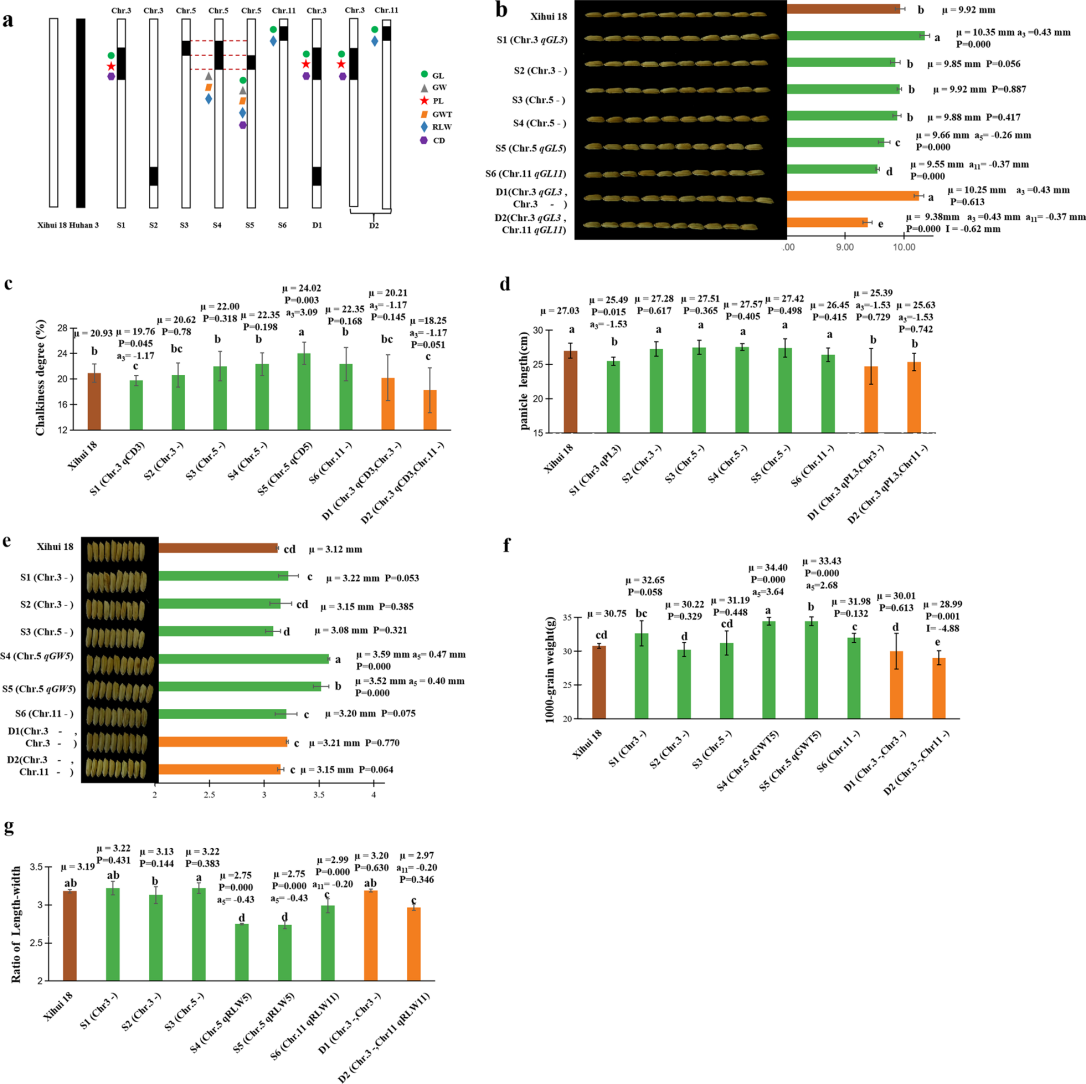
Additive and epistatic effects of QTL for related traits in the SSSLs and DSSLs. **a** Diagram of the locations of substitution segments and QTL in S1–S6 and D1 and D2. **b**–**f** Parameters of QTL in different SSSLs and DSSLs, including grain length (**b**), degree of chalkiness (**c**), panicle length (**d**), grain width (**e**), 1000-grain weight (**f**), and ratio of grain length to width (**g**). Different lowercase letters indicate a significant difference (*P* < 0.05), as determined by Duncan’s multiple comparison. μ: the average value of each line; ai: additive effect for each QTL controlling the trait, whose positive value shows allele from substitution segment increasing phenotypic value, while negative value decreasing one. I: epistatic effect between QTL. *P* < 0.05in SSSL indicates that a QTL existed in the substitution segment of the SSSL, as determined by one-way ANOVA and LSD multiple comparison with Xihui 18; *P* < 0.05 in DSSL indicates that an epistatic effect of Q1 × Q2 existed in DSSL, as detected by two-way ANOVA. S1 (Chr. 3 RM3417 (6.1 Mb)–RM3766 (7.4 Mb)-RM14809 (11.7 Mb)–RM7425 (13.0 Mb)); S2 (Chr. 3 RM5813 (35.2 Mb)–RM3346 (37.6 Mb)–RM1221 (40.1 Mb)); S3 (Chr. 5 RM2010 (1.2 Mb)–RM405 (3.3 Mb)–RM5874 (3.7 Mb)); S4 (Chr. 5 RM2010 (1.2 Mb)–RM405( 3.3 Mb)-RM5874 (3.7 Mb)-RM3322 (4.4 Mb)–RM3328 (5.6 Mb)); S5 (Chr. 5 RM405 (3.3 Mb)–RM5874 (3.7 Mb)-RM3322 (4.4 Mb)–RM3328 (5.6 Mb)); S6 (Chr. 11 RM26038 (1.3 Mb)–RM26045 (1.6 Mb)-RM1812 (2.2 Mb)-RM26114 (2.8 Mb)–RM6085 (3.0 Mb)); D1 (Chr. 3 RM3417 (6.1 Mb)–RM3766 (7.4 Mb)-RM14809 (11.7 Mb)–RM7425 (13.0 Mb), Chr.3 RM5813 (35.2 Mb)–RM3346 (37.6 Mb)–RM1221 (40.1 Mb)); D2 (Chr. 3 RM3417 (6.1 Mb)–RM3766 (7.4 Mb)-RM14809 (11.7 Mb)–RM7425 (13.0 Mb), Chr. 11 RM26038 (1.3 Mb)–RM26045 (1.6 Mb)-RM1812 (2.2 Mb)-RM26114 (2.8 Mb)–RM6085 (3.0 Mb)); the internal markers connected with hyphens indicate the substitution segment from the donor, whereas the markers at each end of the substitution segment linked with ‘–’ indicate that segment recombination might have occurred

Based on the results of primary QTL mapping, we developed six SSSLs (S1–S6) and two DSSLs (D1 and D2) in the F_3_ population by marker-assisted selection (MAS). Among these, S3, S4, and S5 are SSSLs with overlapping substitution segments (Fig. 4a).

Six QTL (*qPL3*, *qGW5*, *qGL11*, *qRLW5*, *qRLW11*, and *qGWT5*) were identified in the SSSLs (Fig. 4a–f), indicating that these QTL are inherited stably. *qBRR11* was not detected in S6, suggesting that the genetic effect of some minor QTL might be influenced by the environment; this QTL contributed only 7.00%. In addition, four QTL (*qGL3*, *qGL5, qCD3*, and *qCD5*) for grain length and degree of chalkiness were detected in S1 and S5 (Fig. 4b, c; Additional file 1) but were not detected in the secondary F_2_ population (Table 1). The reason for this might be genetic noise from the other three substitution segments in F_2_ individuals, which was cancelled out by SSSLs, meaning that QTL were detected more efficiently in SSSLs than in F_2_ plants.

In this section, the positive or negative additive effect (a) of a QTL detected in substitution line indicates increasing or decreasing phenotypic value for the allele from Huhan3 substitition segment compared with recipient Xihui18.

The grain lengths (9.66 and 9.55 mm, respectively) of S5, carrying *qGL5* (a = − 0.26), and S6, carrying *qGL11* (a = − 0.37), were significantly shorter than that of Xihui 18 (9.92 mm). By contrast, the grain length (10.35 mm) of S1, carrying q*GL3* (a = 0.43), was significantly longer than that of Xihui 18 (9.92 mm), and the grain lengths of S2–S4, which lack QTL for grain length, were not significantly different from that of Xihui 18 (Fig. 4b; Additional file 1).

The degree of chalkiness (19.76%) of S1, harboring q*CD3* (a = − 1.17), was significantly lower than that of Xihui 18 (20.93%), while the degree of chalkiness (24.02%) of S5, carrying *qCD5* (a = 3.09), was significantly higher than that of Xihui 18 (20.93%). The degree of chalkiness of the other SSSLs (S2–S4 and S6) without QTL for this trait were not significantly different from that of Xihui 18 (Fig. 4c; Additional file 1).

The panicle length (25.49 cm) of S1 carrying *qPL3* (a = − 1.53) was significantly shorter than that of Xihui 18 (27.03 cm), whereas the panicle lengths of S2–S6, which lacked QTL for this trait, were not significantly different from that of Xihui 18 (Fig. 4d; Additional file 1).

The grain widths (3.59 and 3.52 mm, respectively) of S4 and S5, harboring *qGW5* (a = 0.47 and a = 0.40, respectively), were significantly larger than that of Xihui 18 (3.12 mm), while the grain widths of S1–S3, and S6, which lack QTL for grain width, were not significantly different from that of Xihui 18. *qGW5* was localized to the same substitution interval (RM405–RM5874-RM3322–RM3328) of chromosome 5, based on substitution mapping (Fig. 4e; Additional file 1).

The 1000-grain weights (34.40 and 33.43 g, respectively) of S4 and S5, containing *qGWT5* (a = 3.64 and a = 2.68, respectively), were significantly larger than that of Xihui 18 (30.75 g), whereas the 1000-grain weights of the other SSSLs (S1–S3 and S6), which lack QTL for this trait, were not significantly different from that of Xihui 18. *qGWT5* was localized to the same substitution interval of chromosome 5 as *qGW5* (Fig. 4f; Additional file 1).

The ratios of length to width (2.75, 2.75, and 2.99, respectively) of S4 and S5, carrying *qRLW*5 (both a = − 0.43), and S6, harboring *qRLW11* (a = − 0.20), were significantly lower than that of Xihui 18 (3.19). By contrast, the ratios of length to width of S1–S3, which lack a QTL for this trait, were not significantly different from that of Xihui 18 (Fig. 4g; Additional file 1).

Pyramiding of *qGL3* (a = 0.43) and *qGL11* (a = − 0.37) yielded an epistatic effect of − 0.62, which resulted in a 0.56 mm reduction in grain length in D2. Pyramiding of *qGL3* and *qGL11* resulted in shorter grains than S6 (containing *qGL11*) (Table 2, Fig. 4b; Additional file 2), indicating that *qGL11* is epistatic to *qGL3* (Table 2). However, *qGL3* (a = 0.43) and a substitution locus without a QTL for grain length on chromosome 3 in D1 showed independent inheritance. The grain length of D1 (10.25 mm) was not significantly different from that of S1 (10.35 mm), whereas these grains were significantly longer than those of Xihui 18 and S2 (Table 2, Fig. 4b, Additional file 2). Pyramiding two substitution loci without QTL for 1000-grain weight on chromosomes 3 and 11 in D2 produced an epistatic effect of − 4.88, resulting in a 4.88 g decrease in 1000-grain weight in D2. Thus, the 1000-grain weight of D2 (28.99 g) was significantly lower than that of S1, S6, and Xihui 18 (32.65, 31.98, and 30.75 g, respectively) (Fig. 4f; Additional file 2). All the other QTL in D1 and D2 were independently inherited (Table 2; Fig. 4c, d, e, g; Additional file 2).

**Table 2:** Epistasis between QTLs in DSSL detection by two-way ANOVA

ANOVA	Model term			Substitution line
Trait	Q1	Q2	Q1 × Q2	
Grain length (mm)	*qGL3* (*P* = 0.000)	– (*P* = 0.613)	*qGL3* × – (*P* = 0.613)	D1
	*qGL3* (*P* = 0.000)	*qGL11* (*P* = 0.000)	*qGL3* × *qGL11* (*P* = 0.000)	D2
Chalkiness degree (%)	*qCD3* (*P* = 0.045)	– (*P* = 0.755)	*qCD3* × – (*P* = 0.145)	D1
	*qCD3* (*P* = 0.001)	– (*P* = 0.950)	*qCD3* × – (*P* = 0.051)	D2
Panicle length (cm)	*qPL3* (*P* = 0.002)	– (*P* = 0.886)	*qPL3* × – (*P* = 0.729)	D1
	*qPL3* (*P* = 0.001)	– (*P* = 0.125)	*qPL3* × – (*P* = 0.742)	D2
Grain width (mm)	– (*P* = 0.053)	– (*P* = 0.538)	– × – (*P* = 0.770)	D1
	– (*P* = 0.416)	– (*P* = 0.679)	– × – (*P* = 0.064)	D2
grain weight (g)	– (*P* = 0.388)	– (*P* = 0.122)	– × – (*P* = 0.613)	D1
	– (*P* = 0.062)	– (*P* = 0.181)	– × – (*P* = 0.001)	D2
Ratio of length to width	– (*P* = 0.152)	– (*P* = 0.283)	– × – (*P* = 0.630)	D1
	– (*P* = 0.709)	*qRLW11* (*P* = 0.000)	– × *qRLW11* (*P* = 0.001)	D2

“–” indicates no significant QTL in the according substitution segment of DSSL. *P* < 0.05 indicates additive effects of QTL or epistatic effects between QTLs existed in substitution segments of DSSL; while *P* > 0.05 indicates no additive effects of QTL or no epistatic effects between QTLs existed in substitution segments of DSSL

### Substitution Mapping and Candidate Gene Analysis of ***qGL11***

**Fig. 5:**
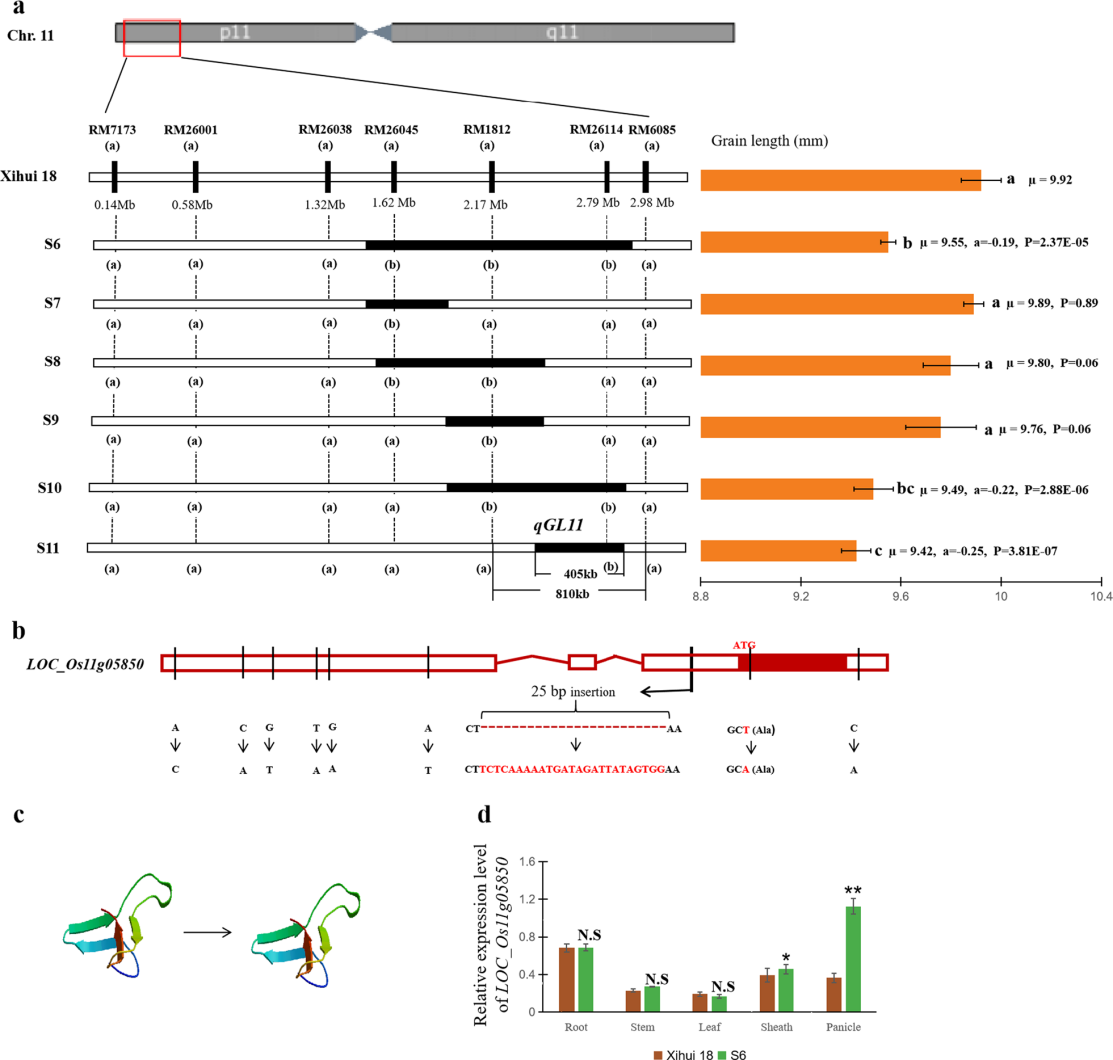
Substitution mapping, sequence analysis, and relative expression level of *qGL11* in Xihui 18 versus S6. **a** Substitution mapping of *qGL11*. Black regions indicate the estimated length of the substitution segment. **b** DNA sequence of *CycT1;3* in S6 compared with Xihui 18. In the candidate gene sequence, the red box represents the coding sequence, the white boxes represent the 5′ UTR and 3′ UTR, the solid red lines represent introns, the black line in the gene sequence represents the mutation site, and the arrow represents a sequence change from Xihui18 to S6. **c** Protein structure of CycT1;3 predicted by SWISS-MODEL. **d** Relative expression levels of the candidate gene *CycT1;3* in root, stem, leaf, sheath, and panicle tissue of Xihui 18 versus S6

Based on the above results, we further analyzed *qGL11* using S6, whose estimated and maximum substitution lengths were 1.42 Mb and 1.66 Mb, respectively (Fig. 5a). For fine mapping of *qGL11*, we developed five novel secondary SSSLs (S7–S11) by crossing Xihui 18 with S6. Based on substitution mapping, *qGL11* was delimited to an estimated substitution interval of 405 kb and a maximum length of 810 kb (Fig. 5a). Ninety genes were identified in this interval of 810 kb, including 40 genes with specific functional descriptions and 50 genes with poorly elucidated functions, such as expressed protein, or retrotransposon protein. Among 40 genes, *LOC_Os11g05850* (*CycT1;3*) was selected as the candidate gene for *qGL11* based on the signaling pathway regulating grain size (Li and Li 2016) and observations of the cell morphology of Z414 glumes. A comparison of the DNA sequences of Xihui 18 with S6 revealed six single nucleotide polymorphisms (SNPs) and a 25-base pair insertion in the 5′ untranslated region (UTR), one SNP in the 3′ UTR, and one SNP in the coding sequence that did not cause an amino acid change (Fig. 5b). Furthermore, the protein structure of CycT1;3 was predicted by SWISS-MODEL (https:/swissmodel.expasy.org/) (Arnold et al, 2006). There was no difference between S6 and Xihui 18 (Fig. 5c). However, the expression levels of *LOC_Os11g05850* were significantly higher in the sheaths and panicles of S6 than in Xihui 18 (Fig. 5d). Thus, this suggests that *LOC_Os11g05850* (*CycT1;3*) might be the gene responsible for *qGL11*.

## Discussion

Restorer lines are important for breeding hybrid rice varieties. Xihui 18 is an elite *indica* rice restorer line developed by Southwest University in Chongqing, China. This variety has many advantages, such as good combining ability, large panicles, multiple grains per panicle, and long, narrow grains. In this study, we constructed the novel CSSL Z414, with short, wide grains, in the Xihui 18 genetic background. Z414 carries four chromosome substitution segments from the donor Huhan 3. None of these substitution segments contained the fertility restoration genes *Rf1*, *Rf2*, *Rf3*, or *Rf4* (Akagi et al. 2004; Itabashi et al. 2011; Cai et al. 2013), indicating that Z414 is restorative. Z414 has seven traits that are different from Xihui 18: it has short, wide, larger grains; shorter panicles; higher brown rice rate; and a greater degree of chalkiness. Z414 is not suitable for use as a rice restorer line because of its high degree of chalkiness. However, its near isogenic background makes it an ideal material for genetic analysis.

Through genetic dissection, we obtained six novel SSSLs (S1–S6) and two DSSLs (D1, D2) harboring the target QTL. Compared to Xihui 18, S1 carries *qGL3* and *qCD3* and exhibits both long grains and a lower degree of chalkiness. D2 harbors *qGL3, qGL11* and *qCD3*, where *qGL11* is epistatic to *qGL3*, and has shorter grains and a lower degree of chalkiness. S4 contains *qGW5*, *qRLW5* and *qGWT5*; it has long, wide, large grains and the same degree of chalkiness as Xihui 18. S6 carries *qGL11* and *qRLW11* and has short, narrow grains and the same degree of chalkiness as Xihui 18. Low chalkiness improves rice grain quality (Yang et al. 2021).

It is important to breed rice cultivars with different grain sizes based on consumer preferences (Feng et al. 2021; Liang et al. 2021). These four SSSLs could potentially be used as restorer lines to breed novel hybrid rice varieties. S5, with short, wide grains (*qGL5*, *qGW5*, *qGWT5*, *qRLW5*) and a high degree of chalkiness (*qCD5*), could be used to study the mechanisms underlying the formation of these traits. Zhang (2021) argued that SSSLs are helpful for rapidly screening traits hidden in the genomes of different donors, making a large amount of previously unexplored genetic variation rapidly available to plant breeders and geneticists, and making the genetic variation directly usable for breeding. SSSLs represent a new resource that could greatly enrich conventional rice breeding (Zhang 2021). The novel SSSLs identified in this study represent an unusual gene pool for genetic research on grain quality and for rice breeding. These SSSLs should be important in allele discovery and research on rice breeding by design.

### *qGL11*, *qRLW11*, *qCD3* and *qBRR11* might be Novel QTL

To further explore the QTL, we developed a secondary F_2_ segregation population from a cross between Xihui 18 and Z414 and generated six SSSLs (S1–S6) and two DSSLs (D1, D2). Twelve QTL were responsible for the seven traits that differed between Z414 and the parental line. Based on analysis of these QTL, the short grains of Z414 were controlled by *qGL11*, *qGL3* and *qGL5*, which were identified in S6, S1 and S5, respectively. The wide grains of Z414 were explained by *qGW5*, which was validated by examining the phenotypes of S4 and S5. The large grains of Z414 were attributed to *qGWT5* (verified by examining S4 and S5). The short panicles of Z414 were explained by *qPL3* (validated by examining S1). The higher brown rice rate of Z414 was explained by *qBRR1*. *qCD5* and *qCD3* (detected in S5 and S1) accounted for the higher degree of chalkiness of Z414.

*OsAPC6* and *OspPLAIIIα* can be acted as candidate genes for *qPL3* based on their physical locations and biological functions*. OsAPC6* interferes with the gibberellin signaling pathway and leads to reduced cell size (Awasthi et al. 2012). *OspPLAIIIα* encodes glycoprotein-related phospholipase A and reduces panicle length (Liu et al. 2015b). *qGW5* for grain width, *qGL5* for grain length*, qRLW5* for the ratio of length to width, and *qGWT5* for 1000-grain weight are located within the interval of RM405 to RM17984 on chromosome 5. *GS5* and *OsTAR1* are potential candidate genes of these QTL based on analysis of the substitution interval. *GS5* positively regulates grain size, as grain width and weight are correlated with its expression level (Xu et al. 2015). *OsTAR1* encodes tryptophan aminotransferase, an IAA biosynthesis gene that regulates the production of IAA in the developing rice grain, together with *OsYUC9* and *OsYUC11* (Abu-Zaitoon et al. 2012)*. OsYUC11*-mediated auxin biosynthesis is essential for endosperm development (Xu et al. 2021).

*qGL3* is located in a similar region to *PGL1*, *OsLG3*, *TUD1* and *OsOFP19*. *PGL1* encodes an atypical basic helix-loop-helix protein that does not bind DNA. Overexpressing *PGL1* increases grain length (Heang and Sassa 2012). *OsLG3* positively regulates grain length, but has no effect on grain quality (Yu et al. 2017). *TUD1* encodes an E3 ubiquitin ligase of the U-box family, which participates in the BR response and interacts with the heterotrimeric G protein subunit D1 to regulate BR-mediated plant growth (Hu et al. 2013). *OsOFP19*, *OSH1*, and *DLT* might form a functional complex that regulates cell proliferation and cell growth (Yang et al. 2018).

*qCD5* is located in a similar region to *Chalk5. Chalk5* encodes a vacuolar H+-translocating pyrophosphatase that increases the chalkiness of the endosperm by disturbing the pH homeostasis of the endomembrane trafficking system in developing seeds (Li et al. 2014). Although some of these genes have been cloned, compared with their identification in mutants, such genes will become more useful in breeding once they have been identified in SSSLs. Consequently, these alleles are important for both biodiversity research and pyramid breeding based on the genetic background of Xihui 18. To the best of our knowledge, *qGL11*, *qRLW11*, *qCD3* and *qBRR11* have not been previously reported. These QTL could be used for fine mapping, cloning, and functional analysis to explore the genetic mechanisms underlying these traits.

### *qGL11* Might be a Novel QTL Encoding CycT1;3, Which has a Previously Unknown Function in Regulating Grain Length

Elucidating the molecular mechanism underlying grain size is important for rice breeding by design. Here, through substitution mapping of *qGL11* using six SSSLs (S6–S11) with overlapping substitution segments, *qGL11* was delimited to an 810-kb maximum substitution interval on chromosome 11. This interval contains 90 genes, including 40 genes whose functions have been described, and 50 genes whose functions were not well elucidated. Among the various genes associated with grain size that have been cloned to date, most are involved in phytohormone pathways, G protein signaling pathways, the ubiquitin–proteasome pathway, the MAPK signal transduction pathway, and pathways regulated by transcription factors (Li and Li 2016). Among the 40 candidate genes, only *CycT1;3* was identified as a candidate gene for *qGL11*. DNA sequencing and real time quantitative PCR analysis of Xihui 18 compared with S6 revealed differences in both the DNA sequences and gene expression levels of *CycT1;3* in sheaths and panicles, indicating that *CycT1;3* might be a strong candidate for *qGL11. CycT1;3* encodes a cyclin protein involved in progression of the cell cycle during mitosis. Cytological analysis showed that the shorter grains of Z414 are caused by a decrease in cell number in glumes rather than a decrease in cell length, suggesting that *qGL11* is related to the cell cycle during meiosis. There have been many studies on cell cycle regulation in yeast and animals, but few in plants. Cell proliferation in plants is mainly controlled by a superfamily of cyclin-dependent kinases. Whereas A-, B-, D-, and E-type cyclins have been described in plants (Nieduszynski et al. 2002), there are few reports on T-type cyclins. Therefore, T-type cyclins in plants would be worth studying in the future.

Qi et al. (2012) reported that *GL3.1* encodes OsPPKL1, which directly uses Cyclin-T1;3 as a substrate to phosphorylate Cyclin-T1;3; the downregulation of the *Cyclin-T1;3* gene in rice resulted in shorter grains. However, how *CycT1;3* affects grain development remains unknown. In this study, although we detected *qGL3* in S1 and found that *qGL11* is epistatic to *qGL3*, *OsPPKL1* was not localized to the same interval in S1. Thus, *qGL11* might be a novel QTL. These results lay the foundation for the in-depth study of *qGL11*.

## Conclusions

We constructed the novel short, wide grain rice CSSL Z414, which carries four substitution segments in the genetic background of *indica* restorer line Xihui 18. Z414 displays seven traits that are different from those of Xihui 18; namely, differences in grain length, grain width, ratio of length to width, 1000-grain weight, brown rice rate, degree of chalkiness, and panicle length. We identified 11 QTL associated with these seven traits in Z414, which were identified in six novel SSSLs (S1–S6) and two novel DSSLs (D1, D2). The short grain trait of Z414 is controlled by *qGL11*, *qGL3* and *qGL5.* Cytological analysis, DNA sequencing, and real time quantitative PCR analysis indicated that *qGL11* might be *CycT1;3*, whose specific role in regulating grain length was previously unknown, suggesting that *qGL11* might be a novel QTL. In particular, S1 carries *qGL3* and *qCD3* and exhibits both long grains and a lower degree of chalkiness. D2 harbors *qGL3, qGL11* and *qCD3*, where *qGL11* is epistatic to *qGL3*, and thus displays shorter grains and a lower degree of chalkiness. These substitution lines could be directly used as restorer lines to breed novel hybrid rice varieties.

## Materials and Methods

### Plant Materials

Z414 was developed from Xihui 18 as the recipient parent and Huhan 3 as the donor parent. Xihui 18 is an elite *indica* rice restorer line bred by Southwest University, with characteristics including good combining ability, large panicles and multiple grains, and narrow, long grains. Huhan 3 is a *japonica* variety, with strong stress resistance and short, wide grains. To identify polymorphisms between Xihui 18 and Huhan 3, 429 simple sequence repeat (SSR) markers covering the whole rice genome were used. Of these, 241 polymorphic markers were then selected to develop CSSLs beginning in the BC_2_F_1_ generation; 20 plants per line were selected in each generation. CSSL Z414, a line with short, wide grains with four substitution segments, was identified in the BC_3_F_7_ population. Chromosome substitution segments were identified as described by Ma et al. (2019), and the estimated lengths of the chromosome substitution segments were calculated as described by Paterson et al. (1991).

### Rice Planting and Cultivation

Xihui 18 was crossed with Z414 to obtain hybrid seeds at the experimental station of Southwest University, Chongqing, China, in July 2018. The hybrid seeds were planted at the Lingshui base in Hainan Province in September of the same year, and the F_1_ seeds were harvested. On March 10, 2019, seeds of Z414, Xihui 18, and the F_2_ population of 150 plants were sown at the experimental station of Southwest University. Thirty seedlings of each parental line and all F_2_ individuals were transplanted to the field on April 20, with 26.4 cm spacing between rows, 16.5 cm between hills, and 10 plants per row. On March 12, 2020, eight individuals were selected from the F_2_ population to develop SSSLs and DSSLs. These plants, as well as Z414 and Xihui 18, were transplanted at the experimental station of Southwest University, using 30 plants per line. On March 10, 2021, six SSSLs and two DSSLs together with Xihui 18 and Z414 were planted in Chongqing, again with 30 individuals transplanted per line. Conventional management practices were applied.

### Assessment of Agronomic Traits and Quality Parameters

During the maturation period, 10 plants each of Xihui 18 and Z414, the six SSSLs, and the two DSSLs were harvested, together with 150 individuals of the F_2_ population. Eleven yield-related traits were investigated: plant height, panicle number per plant, panicle length, spikelet number per panicle, grain number per panicle, seed-setting rate, grain length, grain width, ratio of length to width, 1000-grain weight, and yield per plant. The specific methods followed Wang et al. (2020).

Five quality parameters were analyzed, referring to the national standard GB/T5495-2008. First, 10 g grain tissue from Xihui 18, Z414, the 150 F_2_ individuals, six SSSLs, and two DSSLs were ground into brown rice. The brown rice was milled into polished rice using a CLS.JNM-1 rice husker, and the brown rice rate and head rice rate were calculated. The chalky rice rate and degree of chalkiness were measured using all head rice for each sample with a Wanshen SC-E instrument. The gel consistency was measured as described in Tang et al. (1991).

The mean values of each trait were used for further analysis.

### Scanning Electron Microscopy of Z414 and Xihui 18 Glumes

Following the completion of the booting stage and before the heading period, the inner and outer epidermal cells of Xihui 18 and Z414 glumes were examined under a Hitachi SU3500 scanning electron microscope (Hitachi, Tokyo, Japan) with a frozen stage (− 40 °C) under vacuum.

### QTL Mapping

The QTL mapping population was a secondary F_2_ population comprising 150 individuals derived from crosses between Xihui 18 and Z414. The improved cetyltrimethylammonium bromide (CTAB) method described by McCouch et al. (1988) was used to extract DNA from the parents and 150 F_2_ individuals. PCR amplification, polyacrylamide gel electrophoresis, and rapid silver staining were carried out as described by Zhao et al. (2016). Xihui 18 lanes were scored as “a”, Z414 lanes were scored as “b”, heterozygous lanes were scored as “h”, and the absence of marker lanes was scored as “u”. Based on the lanes of each marker located in the substitution segment of Z414, together with the phenotypic value of individuals of the F_2_ population, Interval Mapping (IM) with MapQTL 6.0 (Van Ooijen 2009) was used to identify QTL. The LOD threshold of significant QTL was calculated by a permutation test with a genome-wide significance level of *P* < 0.05 and permutation tests = 1000. The QTL with a LOD > 2.5. The determination of QTL depended on the highest peak LOD and the directions of additive effects. The positive genetic effect of each QTL indicated that the allele of Huhan 3 substitution segment of Z414 increased the phenotypic value, whereas the negative effect indicated that the allele of Xihui18 in substitution interval increased the phenotypic value.

### Development of SSSLs and DSSLs

Based on the results of QTL mapping in 2019, eight individuals with the target segment and few heterozygous markers were selected from the F_2_ population and used to develop SSSLs and DSSLs by MAS. Individuals were planted as a single line (Z728–Z735) in 2020. The leaves of 20 individuals per line were collected and used to extract DNA for genotyping for MAS using both the target substitution markers and residual heterozygous markers. The SSSLs and DSSLs were developed based on the rule that each substitution line carried only the homozygous target substitution segment, while the lanes of other markers were same as those of Xihui 18.

Ten plants each of the six SSSLs, two DSSLs, and Xihui 18 were harvested from each plot after maturity in August 2021. All traits were measured again using the same methods as in 2019.

### Validation of Target QTL in the SSSLs

Each SSSL_i_ (S1–S6) was given the hypothesis (H0) that no QTL existed in the substitution segment of the SSSL_i_. When the *P* value was less than 0.05 according to one-way analysis of variance (ANOVA) and LSD multiple comparison with Xihui 18 by each SSSL_i_ in IBM SPSS Statistics 23.0, we denied the hypothesis and considered that a QTL for a certain trait existed in the SSSL_i_. According to the genetic model, in a certain environment (same year and same experimental field and no replicate plot designed) P_0_ = μ_0_ + ε for Xihui 18 and P_i_ = μ_0_ + a_i_ + ε for an SSSL carrying a specific QTL, where P_0_ and P_i_ represent the phenotypic value of any plant in a plot of Xihui 18 and the SSSL_i_. μ_0_ represents the mean value of the Xihui 18 population, a_i_ represents the additive effect of the QTL from substitution segment of donor Huhan3, whose positive effect indicates increasing phenotypic value and negative one shows decreasing phenotypic value in substitution lines, and ε represents residual error.

Thus, the additive effect of the QTL was calculated as the difference between the mean phenotypic values of the SSSL and Xihui 18 (Liang et al. 2021). All calculations were conducted using Microsoft Excel 2016.

### Analysis of Epistatic Interactions Between Target QTL in the DSSLs

For each DSSL_ij_, the hypothesis (H0) that two loci (Q1 and Q2) for a certain trait located in the “i” and “j” substitution segments were independently inherited was expressed as “2 + 0 = 1 + 1”. When the *P* value was greater than 0.05 for Q1 × Q2 using two-way ANOVA in IBM SPSS Statistics 23.0, we accepted the hypothesis that Q1 and Q2 in DSSL_ij_ were independently inherited. At this time, the phenotypic value of (Xihui 18 + DSSL_ij_) was the same as that of (SSSL_i_ + SSSL_j_). By contrast, when the *P* value was less than 0.05 for Q1 × Q2, we denied the hypothesis and considered that an epistatic interaction occurred between the two allelic loci Q1 and Q2, namely “2 + 0 ≠ 1 + 1”. According to the genetic models, P_0_ = μ_0_ + ε for Xihui18, P_i_ = μ_0_ + a_i_ + ε for SSSL_i,_ P_j_ = μ_0_ + a_j_ + ε for SSSL_j_ and P_ij_ = μ_0_ + a_i_ + a_j_ + I_ij_ + ε for DSSL_ij_, where P_ij_ represents the phenotypic value of any plant in a plot of the DSSL_ij_; μ_0_ represents the mean value of the Xihui 18 population; a_i_ and a_j_ represent the additive effect of QTL in substitution segment i and j, respectively; and I_ij_ represents the a_i_a_j_ epistatic effect between QTL in substitution segment i and j. Thus, the epistatic effects between non-allele QTL were the difference between the mean phenotypic values of (Xihui 18 + DSSL_ij_) and (SSSL_i_ + SSSL_j_) (Liang et al. 2021). Finally, we used IBM SPSS Statistics 23.0 to conduct multiple comparisons of all SSSLs and DSSLs as well as Xihui 18.

### Overlapping Substitution Mapping and Candidate Gene Analysis of *qGL11*

To develop secondary SSSLs for *qGL11*, S6 was crossed with Xihui 18 in 2020. Five SSSLs (S7–S11) with overlapping substitution segments were developed from the cross in 2021. The maximum and estimated substitution length of the secondary SSSLs were estimated based on the marker positions (Paterson et al. 1991). QTL were located by substitution mapping (Yang et al. 2021). When grain length significantly differed between a secondary SSSL and Xihui 18, a QTL for grain length was detected in the substitution segment of the SSSL. When multiple substitution segments in SSSLs with the target trait overlapped, the QTL was localized to the overlapping region (Yang et al. 2021). The additive effect of the QTL was calculated as half the difference between the mean phenotypic values of the SSSL and Xihui18 (Liang et al. 2021). Within the estimated substitution intervals, we predicted the candidate gene information and combined this information with gene annotations to identify possible candidate genes of *qGL11* using Gramene (http://www.gramene.org/), the Rice Annotation Project Database (https://rapdb.dna.affrc.go.jp/), and the China National Rice Database Center (http://www.ricedata.cn/). The candidate gene sequence was downloaded, including 3000 bp before the start codon (ATG) and 1500 bp after the stop codon. Primers were designed using Vector NTI to amplify the target fragments using Xihui 18 and S6 DNA as templates. The amplicons were sequenced by Tsingke Biological Technology Co., Ltd. (Chongqing, China).

### Total RNA Extraction and Real Time Quantitative PCR Analysis

Total RNA was extracted from root, stem, leaf, sheath, and panicle tissue of Xihui 18 and S6 at the booting stage using an RNAprep Pure Plant RNA Purification Kit (Tiangen, Beijing, China); reverse transcribed using the GoScript Reverse Transcription System; and quantitatively analyzed on a Bio-Rad CYF96 system using Real-time PCR Master Mix (TaKaRa Biotechnology (Dalian, China) Co. Ltd.). The rice gene *Actin* (LOC_Os03g50885) was used as the internal control to normalize all data.

## Supplementary Information

**Additional file 1.** The main results of QTL identification based on Substitution lines (S1–S6; D1–D2) and recipient Xihui 18 by one way ANOVA and LSD multiple comparisons. Between groups represent the variations among all SSSLs (S1–S6), DSSLs (D1 and D2) and recipient Xihui18 comparison for each trait as grain length, chalkiness degree, panicle length, grain width, 1000-grain weight, ratio of length to width. Within groups represent variation for errors. LSD represents least significant difference multiple comparison, in which I VAR0001 is Xihui 18, J VAR0001 is substitution lines (S1–S6 and D1–D2), respectively, Mean difference (I–J) is the difference value between Xihui 18 and each substitution line. Sig. represent probability value (P) for each trait difference between each substitution line and Xihui 18. When Sig. < 0.05 represents a QTL for a certain trait existed in a SSSL (S1–S6) or DSSL.

**Additional file 2.** The main results of epistatic interaction between Q1 (located in i substitution segment) and Q2 (located in j substitution segment) in DSSLs (D1–D2) by two-way ANOVA. Tests of between subjects effects represent Q1, Q2 and Q1× Q2 test using DSSL containing both the i and j substitution segments and the responding SSSLi and SSSLj by two-way ANOVA, in which sig. < 0.05 for Q1 or Q2 indicate additive effects of Q1 or Q2 existed; sig. > 0.05 for Q1 or Q2 indicate no significant additive effects of Q1 or Q2 existed and “-“ was showed; sig. < 0.05 for Q1 × Q2 indicate epistatic effect of Q1 and Q2 interaction existed in the DSSL; sig. >0.05 for Q1 × Q2 indicate independent inheritance of Q1 and Q2 in the DSSL.

## Data Availability

The datasets supporting the conclusions of this article are included within the article.

## Abbreviations

QTL: Quantitative trait locus/loci
CSSL: Chromosome segment substitution lines
SSSL: Single-segment substitution line
DSSL: Dual-segment substitution line
SSR: Simple sequence repeat
MAS: Marker-assisted selection
PL: Panicle length
GL: Grain length
GW: Grain width
RLW: Grain length-to-width ratio
GWT: 1000-Grain weight
BRR: Brown rice rate
CD: Chalkiness degree
SNP: Single nucleotide polymorphism
UTR: Untranslated region
CDS: Coding sequence
