# Supplementary material for: QTL Analysis of Z414, a Chromosome Segment Substitution Line with Short, Wide Grains, and Substitution Mapping of qGL11 in Rice
Source: Rice (N Y). 2022 May 9;15:25. doi: 10.1186/s12284-022-00571-7 (PMC9085999; doi:10.1186/s12284-022-00571-7)
Supplement: Supplementary file 1 — Additional file 1. The main results of QTL identification based on Substitution lines (S1–S6; D1–D2) and recipient Xihui 18 by one way ANOVA and LSD multiple comparisons. Between groups represent the variations among all SSSLs (S1–S6), DSSLs (D1 and D2) and recipient Xihui18 comparison for each trait as grain length, chalkiness degree, panicle length, grain width, 1000-grain weight, ratio of length to width. Within groups represent variation for errors. LSD represents least significant difference multiple comparison, in which I VAR0001 is Xihui 18, J VAR0001 is substitution lines (S1–S6 and D1–D2), respectively, Mean difference (I–J) is the difference value between Xihui 18 and each substitution line. Sig. represent probability value (P) for each trait difference between each substitution line and Xihui 18. When Sig. < 0.05 represents a QTL for a certain trait existed in a SSSL (S1–S6) or DSSL. [file 12284_2022_571_MOESM1_ESM.pdf]

Supplement data 1: One-way ANOVA and LSD multiple comparison for 6 traits between Xihui 18 and S1-S6

Grain length (mm):

ANOVA

	Sum of Squares	df	Mean Square	F	Sig.
Between Groups	4.165	8	0.521	112.797	0.000
Within Groups	0.231	50	0.005		
Total	4.396	58			

Multiple Comparisons

	(I) VAR00001	(J) VAR00001	Mean Difference (I-J)	Std. Error	Sig.	95% Confidence Interval	
						Lower Bound	Upper Bound
LSD	Xihui 18	S1	-0.42833*	0.04114	0.000	-0.511	-0.3457
		S2	0.06925	0.03448	0.056	0.01	0.1485
		S3	-0.00503	0.03508	0.887	-0.0755	0.0654
		S4	0.03367	0.04114	0.417	-0.049	0.1163
		S5	0.25742*	0.0378	0.000	0.1815	0.3333
		S6	0.36747*	0.04386	0.000	0.2794	0.4556
		D1	-0.33387*	0.04804	0.000	-0.4304	-0.2374
		D2	0.54043*	0.03669	0.000	0.4667	0.6141

Chalkiness degree (%):

ANOVA

	Sum of Squares	df	Mean Square	F	Sig.
Between Groups	278.326	8	34.791	5.837	0
Within Groups	530.518	89	5.961		
Total	808.844	97			

Multiple Comparisons

	(I) VAR00001	(J) VAR00001	Mean Difference (I-J)	Std. Error	Sig.	95% Confidence Interval	
						Lower Bound	Upper Bound
LSD	Xihui 18	S1	1.17193*	1.06676	0.045	-0.9477	3.2915
		S2	0.30758	1.09737	0.78	-1.8729	2.488
		S3	-1.07209	1.06676	0.318	-3.1917	1.0475
		S4	-1.42242	1.09737	0.198	-3.6029	0.758
		S5	-3.08742*	1.01914	0.003	-5.1124	-1.0624
		S6	-1.41659	1.01914	0.168	-3.4416	0.6084
		D1	0.71758	1.01914	0.483	-1.3074	2.7426
		D2	2.67937*	1.00021	0.009	0.692	4.6668

Panicle length (cm)：

ANOVA

	Sum of Squares	df	Mean Square	F	Sig.
Between Groups	66.699	8	8.337	6.222	0
Within Groups	73.701	55	1.34		
Total	140.4	63			

Multiple Comparisons

	(I) VAR00001	(J) VAR00001	Mean Difference (I-J)	Std. Error	Sig.	95% Confidence Interval	
						Lower Bound	Upper Bound
LSD	Xihui 18	S1	1.53380*	0.64568	0.021	0.2398	2.8278
		S2	-0.2522	0.50197	0.617	-1.2582	0.7538
		S3	-0.48627	0.53188	0.365	-1.5522	0.5796
		S4	-0.5417	0.64568	0.405	-1.8357	0.7523
		S5	-0.39803	0.58337	0.498	-1.5671	0.7711
		S6	0.5715	0.69563	0.415	-0.8226	1.9656
		D1	1.63860*	0.77173	0.038	0.092	3.1852
		D2	1.40755*	0.56249	0.039	1.2803	3.5348

Grain width (mm)：

ANOVA

	Sum of Squares	df	Mean Square	F	Sig.
Between Groups	1.65	8	0.206	44.329	0
Within Groups	0.237	51	0.005		
Total	1.887	59			

Multiple Comparisons

	(I) VAR00001	(J) VAR00001	Mean Difference (I-J)	Std. Error	Sig.	95% Confidence Interval	
						Lower Bound	Upper Bound
LSD	Xihui 18	S1	-0.095	0.04402	0.053	-0.1834	-0.0066
		S2	-0.0295	0.03366	0.385	-0.0971	0.0381
		S3	0.03533	0.03522	0.321	-0.0354	0.106
		S4	-0.47200*	0.0413	0	-0.5549	-0.3891
		S5	-0.39904*	0.03794	0	-0.4752	-0.3229
		S6	-0.08	0.04402	0.075	-0.1684	0.0084
		D1	-0.08557	0.04402	0.057	-0.174	0.0028
		D2	-0.03951	0.03794	0.303	-0.1157	0.0367

1000-grain weight (g)：

ANOVA

	Sum of Squares	df	Mean Square	F	Sig.
Between Groups	150.101	8	18.763	14.036	0
Within Groups	69.511	52	1.337		
Total	219.612	60			

Multiple Comparisons

	(I) VAR00003	(J) VAR00003	Mean Difference (I-J)	Std. Error	Sig.	95% Confidence Interval	
						Lower Bound	Upper Bound
LSD	Xihui 18	S1	-1.93946	0.67699	0.058	-3.2479	-0.531
		S2	0.53437	0.54202	0.329	-0.5533	1.622
		S3	-0.43596	0.56977	0.448	-1.5793	0.7074
		S4	-3.64286*	0.67699	0	-5.0013	-2.2844
		S5	-2.67614*	0.618	0	-3.9163	-1.436
		S6	-1.22052	0.79784	0.132	-2.8215	0.3805
		D1	-0.74852	0.79784	0.11	-2.8995	0.3025
		D2	1.76377*	0.59838	0.006	0.523	2.9245

Ratio of Length to width：

ANOVA

	Sum of Squares	df	Mean Square	F	Sig.
Between Groups	1.825	8	0.228	46.828	0
Within Groups	0.239	49	0.005		
Total	2.064	57			

Multiple Comparisons

	(I) VAR00003	(J) VAR00003	Mean Difference (I-J)	Std. Error	Sig.	95% Confidence Interval	
						Lower Bound	Upper Bound
LSD	Xihui 18	S1	-0.03504	0.04415	0.431	-0.1238	0.0537
		S2	0.0559	0.03765	0.144	-0.0198	0.1316
		S3	-0.03367	0.03823	0.383	-0.1105	0.0432
		S4	0.43304*	0.04415	0	0.3443	0.5218
		S5	0.43879*	0.04087	0	0.3567	0.5209
		S6	0.19812*	0.04682	0	0.104	0.2922
		D1	-0.01351	0.04682	0.774	-0.1076	0.0806
		D2	0.21341*	0.04087	0	0.1313	0.2955
