# Supplementary material for: QTL Analysis of Z414, a Chromosome Segment Substitution Line with Short, Wide Grains, and Substitution Mapping of qGL11 in Rice
Source: Rice (N Y). 2022 May 9;15:25. doi: 10.1186/s12284-022-00571-7 (PMC9085999; doi:10.1186/s12284-022-00571-7)
Supplement: Supplementary file 2 — Additional file 2. The main results of epistatic interaction between Q1 (located in i substitution segment) and Q2 (located in j substitution segment) in DSSLs (D1–D2) by two-way ANOVA. Tests of between subjects effects represent Q1, Q2 and Q1× Q2 test using DSSL containing both the i and j substitution segments and the responding SSSLi and SSSLj by two-way ANOVA, in which sig. < 0.05 for Q1 or Q2 indicate additive effects of Q1 or Q2 existed; sig. > 0.05 for Q1 or Q2 indicate no significant additive effects of Q1 or Q2 existed and “-“ was showed; sig. < 0.05 for Q1 × Q2 indicate epistatic effect of Q1 and Q2 interaction existed in the DSSL; sig. >0.05 for Q1 × Q2 indicate independent inheritance of Q1 and Q2 in the DSSL. [file 12284_2022_571_MOESM2_ESM.pdf]

Supplement Data2: Two-way ANOVA of two QTL epistattic interaction in substitution segments of each DSSL

Grain length：

D1:

Tests of Between-Subjects Effects

Source	Type III Sum of Squares	df	Mean Square	F	Sig.
Corrected Model	0.944[a]	3	0.315	103.622	0.000
Intercept	2075.737	1	2075.737	683620.973	0.000
qGL3 (S1)	0.863	1	0.863	284.283	0.000
Substitution Seg. (S2)	0.001	1	0.001	0.264	0.613
qGL3(S1)×substitution seg.(S2)	0.001	1	0.001	0.385	0.542
Error	0.064	21	0.003		
Total	2529.360	25			
Corrected Total	1.008	24			

a. R Squared = 0.937 (Adjusted R Squared = 0.928)

D2:

Tests of Between-Subjects Effects

Source	Type III Sum of Squares	df	Mean Square	F	Sig.
Corrected Model	3.214[a]	3	1.071	249.372	0.000
Intercept	2072.625	1	2072.625	482406.828	0.000
qGL3 (S1)	0.088	1	0.088	20.466	0.000
qGL11 (S6)	2.407	1	2.407	560.332	0.000
qGL3(S1)×*qGL11*(S6)	0.487	1	0.487	113.465	0.000
Error	0.082	19	0.004		
Total	2195.523	23			
Corrected Total	3.296	22			

a. R Squared = 0.975 (Adjusted R Squared = 0.971)

Chalkiness degree (%):

D1:

Tests of Between-Subjects Effects

Source	Type III Sum of Squares	df	Mean Square	F	Sig.
Corrected Model	33.286[a]	3	11.095	2.205	0.103
Intercept	17125.141	1	17125.141	3403.234	0.000
qCD3(S1)	21.689	1	21.689	4.310	0.045
Substitution Seg. (S2)	0.497	1	0.497	0.099	0.755
qCD3(S1) ×substitution seg.(S2)	11.130	1	11.130	2.212	0.145
Error	191.217	38	5.032		
Total	17572.906	42			
Corrected Total	224.502	41			

a. R Squared = 0.148 (Adjusted R Squared = 0.081)

D2:

Tests of Between-Subjects Effects

Source	Type III Sum of Squares	df	Mean Square	F	Sig.
Corrected Model	112.157[a]	3	37.386	6.305	0.001
Intercept	18817.033	1	18817.033	3173.565	0.000
qCD3(S1)	79.024	1	79.024	13.328	0.001
Substitution Seg. (S6)	0.024	1	0.024	0.004	0.950
qCD3(S1) ×substitution seg.(S6)	24.348	1	24.348	4.106	0.051
Error	249.031	42	5.929		
Total	19295.816	46			
Corrected Total	361.188	45			

a. R Squared = 0.311 (Adjusted R Squared = 0.261)

Panicle length (cm):

D1:

Tests of Between-Subjects Effects

Source	Type III Sum of Squares	df	Mean Square	F	Sig.
Corrected Model	17.758[a]	3	5.919	4.112	0.016
Intercept	15337.374	1	15337.374	10653.736	0.000
qPL3(S1)	16.258	1	16.258	11.293	0.002
Substitution Seg. (S2)	0.030	1	0.030	0.021	0.886
qPL3(S1) ×substitution seg.(S2)	0.177	1	0.177	0.123	0.729
Error	37.430	26	1.440		
Total	21467.675	30			
Corrected Total	55.188	29			

a. R Squared = 0.322 (Adjusted R Squared = 0.244)

D2:

Tests of Between-Subjects Effects

Source	Type III Sum of Squares	df	Mean Square	F	Sig.
Corrected Model	26.611[a]	3	8.870	7.419	0.001
Intercept	15640.869	1	15640.869	13082.602	0.000
qPL3(S1)	16.551	1	16.551	13.844	0.001
Substitution Seg. (S6)	3.044	1	3.044	2.546	0.125
qPL3(S1) ×substitution seg.(S6)	0.133	1	0.133	0.111	0.742
Error	26.302	22	1.196		
Total	17497.515	26			
Corrected Total	52.913	25			

a. R Squared = 0.503 (Adjusted R Squared = 0.435)

Grain width (mm)：

D1:

Tests of Between-Subjects Effects

Source	Type III Sum of Squares	df	Mean Square	F	Sig.
Corrected Model	0.024[a]	3	0.008	1.455	0.253
Intercept	215.886	1	215.886	39043.578	0.000
Substitution Seg. (S1)	0.023	1	0.023	4.178	0.053
Substitution Seg. (S2)	0.002	1	0.002	0.390	0.538
Substitution Seg.(S1) × substitution seg.(S2)	0.000	1	0.000	0.087	0.770
Error	0.127	23	0.006		
Total	269.420	27			
Corrected Total	0.151	26			

a. R Squared = 0.159 (Adjusted R Squared = 0.050)

D2:

Tests of Between-Subjects Effects

Source	Type III Sum of Squares	df	Mean Square	F	Sig.
Corrected Model	0.019[a]	3	0.006	1.678	0.212
Intercept	192.448	1	192.448	50124.571	0.000
Substitution Seg. (S1)	0.003	1	0.003	0.697	0.416
Substitution Seg. (S6)	0.001	1	0.001	0.178	0.679
Substitution Seg.(S1) × substitution seg.(S6)	0.015	1	0.015	3.975	0.064
Error	0.061	16	0.004		
Total	199.834	20			
Corrected Total	0.081	19			

a. R Squared = 0.239 (Adjusted R Squared = 0.097)

1000-grain weight (g)：

D1:

Tests of Between-Subjects Effects

Source	Type III Sum of Squares	df	Mean Square	F	Sig.
Corrected Model	5.675[a]	3	1.892	1.475	0.246
Intercept	20030.910	1	20030.910	15623.243	0.000
Substitution Seg. (S1)	0.990	1	0.990	0.772	0.388
Substitution Seg. (S2)	3.286	1	3.286	2.563	0.122
Substitution Seg.(S1) × substitution seg.(S2)	0.338	1	0.338	0.263	0.613
Error	30.771	24	1.282		
Total	26242.777	28			
Corrected Total	36.446	27			

a. R Squared = 0.156 (Adjusted R Squared =0 .050)

D2：

Tests of Between-Subjects Effects

Source	Type III Sum of Squares	df	Mean Square	F	Sig.
Corrected Model	31.123[a]	3	10.374	7.951	.001
Intercept	19019.595	1	19019.595	14577.488	.000
Substitution Seg. (S1)	5.123	1	5.123	3.927	.062
Substitution Seg. (S6)	2.522	1	2.522	1.933	.181
Substitution Seg.(S1) × substitution seg.(S6)	18.621	1	18.621	14.272	.001
Error	24.790	19	1.305		
Total	21474.666	23			
Corrected Total	55.913	22			

a. R Squared = 0.557 (Adjusted R Squared = 0.487)

Ratio of Length to width：

D1:

Tests of Between-Subjects Effects

Source	Type III Sum of Squares	df	Mean Square	F	Sig.
Corrected Model	0.035[a]	3	0.012	1.767	0.184
Intercept	218.925	1	218.925	32883.494	0.000
Substitution Seg. (S1)	0.015	1	0.015	2.211	0.152
Substitution Seg. (S2)	0.008	1	0.008	1.215	0.283
Substitution Seg.(S1) × substitution seg.(S2)	0.002	1	0.002	0.239	0.630
Error	0.140	21	0.007		
Total	251.459	25			
Corrected Total	0.175	24			

a. R Squared = 0.202 (Adjusted R Squared = 0.087)

D2:

Tests of Between-Subjects Effects

Source	Type III Sum of Squares	df	Mean Square	F	Sig.
Corrected Model	0.270[a]	3	0.090	26.396	0.000
Intercept	192.893	1	192.893	56634.698	0.000
Substitution Seg. (S1)	0.000	1	0.000	0.145	0.709
qRLW11(S6)	0.252	1	0.252	73.850	0.000
Substitution Seg.(S1) × qRLW11(S6)	0.003	1	0.003	0.938	0.346
Error	0.058	17	0.003		
Total	200.226	21			
Corrected Total	0.328	20			

a. R Squared = 0.823 (Adjusted R Squared = 0.792)
